# Mining Candidate Genes for Maize Tassel Spindle Length Based on a Genome-Wide Association Analysis

**DOI:** 10.3390/genes15111413

**Published:** 2024-10-31

**Authors:** Xudong Cao, Han Lu, Zhiwen Zhao, Yujie Lian, Hui Chen, Mengfan Yu, Fan Wang, Huayue Sun, Dong Ding, Xuehai Zhang, Xiaoyang Chen, Jihua Tang

**Affiliations:** Key Laboratory of Wheat and Maize Crops Science, College of Agronomy, Henan Agricultural University, Zhengzhou 450002, China; caoxudong10466@163.com (X.C.); 13183009952@163.com (H.L.); zzw16692520062@163.com (Z.Z.); lianyujie0405@163.com (Y.L.); 17339301889@163.com (H.C.); 19561300964@163.com (M.Y.); 16637200615@163.com (F.W.); sunhuayue@henau.edu.cn (H.S.); dingdong0216@hotmail.com (D.D.); xuehaizhang@henau.edu.cn (X.Z.); tangjihua1@163.com (J.T.)

**Keywords:** maize, tassel spindle length, genome-wide association analysis, candidate genes

## Abstract

Maize tassel spindle length is closely related to the number of pollen grains and the duration of the flowering stage, ultimately affecting maize yield and adaptations to stress conditions. In this study, 182 maize inbred lines were included in an association population. A genome-wide association study was conducted on maize tassel spindle length using the Q + K model. With *p* ≤ 1.0 × 10^−4^ applied as the significance threshold, 240 SNPs significantly associated with tassel spindle length were detected, which were associated with 99 quantitative trait loci (QTLs), with 21 QTLs detected in two or more environments. Moreover, 51 candidate genes were detected in 21 co-localized QTLs. A KEGG enrichment analysis and candidate gene expression analysis indicated that *Zm00001d042312* affects plant hormone signal transduction and is highly expressed in maize tassels. A haplotype analysis of *Zm00001d042312* revealed three main haplotypes, with significant differences between Hap1 and Hap2. In conclusion, we propose that *Zm00001d042312* is a gene that regulates maize tassel spindle length. This study has further elucidated the genetic basis of maize tassel spindle length, while also providing excellent genetic targets and germplasm resources for the genetic improvement of maize tassel spindle length and yield.

## 1. Introduction

Maize is used as a source of food and feed, making it an important crop for national food security and economic development [1,2]. Continual improvements in living standards have been accompanied by an increasing demand for high-protein foods, such as meat, eggs, and dairy products, which has contributed to the growing demand for maize [3,4]. Tassels located at the top of maize plants significantly affect maize yield because they are involved in fertilization processes and nutrient distribution. And the maize tassel development is easily influenced by environmental factors, such as drought [5], high temperature [6], daily light integral or photoperiod [7]. Spindle length is a critical characteristic related to tassel architecture. An appropriate length increases the number of pollen grains, while also prolonging the pollen shedding period, thereby enhancing maize fertilization rates and reducing the length of ear barren tip under adverse environmental conditions [8,9]. Effective resource allocation plays a significant role in maize yield [10]. An appropriate length can save materials and energy and encourage more of them to flow to the ear. Therefore, unraveling the genetic basis of maize tassel spindle length and identifying favorable alleles is critical for increasing maize productivity.

Maize tassel development is a complex and precisely regulated process that primarily involves the proliferation and differentiation of stem cells [11]. The shoot apical meristem (SAM) at the top of the plant contains pluripotent stem cells and serves as the starting point for plant growth and development. During the transition from the vegetative growth stage to the reproductive growth stage, SAM transforms into the inflorescence meristem (IM), marking the beginning of reproductive growth, after which IM elongates and differentiates to form branch meristems (BMs) that provide the foundation of the tassel structure. Simultaneously, the spikelet pair meristem (SPM) develops at the periphery of IM and BM. An individual SPM can produce a pair of spikelet meristems (SMs), which eventually develop into two floret meristems (FMs) that correspond to the upper and lower flowers [12,13,14].

The importance of phytohormones for maize tassel formation and development has been revealed by several studies. Gallavotti et al. showed that *barren stalk1* (*ba1*) encodes a bHLH transcription factor involved in the transport of auxin in plants, which modulates the initiation of maize inflorescence meristems. Mutations in *ba1* lead to decreases in the number of tassel branches and spikelets [15]. Skirpan et al. reported that *barren inflorescence2* (*bif2*) encodes a serine/threonine protein kinase that regulates auxin transport. Mutations in *bif2* affect the development of axillary meristems, resulting in decreases in the number of tassel spikelets and florets [16]. Gallavotti et al. also showed that *sparse inflorescence1* (*spi1*) is a flavin monooxygenase required during the vegetative and reproductive development in maize. Specifically, it is involved in the synthesis of auxin. A mutation in this gene results in abnormal BMs and SPMs development, leading to the reduction in branch and spikelet number [17]. Phillips et al. demonstrated that *vanishing tassel 2* (*vt2*) encodes a grass-specific tryptophan aminotransferase involved in auxin synthesis. Mutations in *vt2* have detrimental effects on vegetative and reproductive growth stages, ultimately resulting in the abnormal development of tassel [18]. However, the function of cytokinin in tassel development is not well understood.

The complex morphology of the mature maize tassel is usually made up of tassel spindle length, central spike length, tassel branch angle, branch length, tassel weight, and tassel branch number, which are typical quantitative traits regulated by multiple minor effect genes. Xu et al. used 19,838 single nucleotide polymorphism (SNP) markers to conduct high-resolution QTL mapping for five tassel morphological traits (tassel branch angle, tassel branch number, tassel branch length, tassel spindle length, and tassel peduncle length) in 866 BC_2_S_3_ recombinant inbred lines. A total of 72 QTLs were detected for these five tassel traits [19]. Wang et al. used 43,958 high-quality SNP markers to perform multi-model genome-wide association analyses on the tassel branch number of 359 inbred lines and 273 double haploid lines across three environments. A total of 12 QTLs was consistently detected in multiple environments. One of these QTLs, the candidate gene *Zm00001d016615*, was identified as a putative target of the *RA1* gene [10]. In addition, Wu et al. identified six QTLs associated with tassel spindle length using the Y915 and Zheng58 recombinant inbred line (RIL) population. These QTLs were distributed across chromosomes 1, 8, and 10. Moreover, they explained 5.10–14.63% of the phenotypic variance [20]. Gao et al. analyzed tassel spindle length in chromosome segment substitution lines derived from Ye478 and Qi319 across four different environments. 27 QTLs were detected in two or more environments (distributed on chromosomes 1, 2, 3, 4, 5, 7, 8, 9, and 10) and 16 QTLs were detected in three or more environments [21]. Dong et al. examined tassel spindle length using 258 RILs across three environments, identifying 22 QTLs on chromosomes 1, 4, 5, 6, 7, and 8 that explained 4.2–15.84% of the phenotypic variance [22]. Gao et al. performed a genome-wide association study (GWAS) on maize tassel spindle length using 500 natural populations. They identified three significant QTLs on chromosomes 2 and 5 that explained 5.04–6.34% of the phenotypic variance [23]. In another GWAS, Pan et al. identified 13 tassel spindle length-related QTLs, mainly on chromosomes 1, 7, 8, and 10 [24]. Yang et al. determined the heritability of tassel spindle length in 513 maize inbred lines (heritability of 0.683) and identified 14 significant QTLs based on a GWAS [25]. Yi et al. analyzed F_2:3_ families derived from 08-641 and Ye478, as well as a population comprising 301 RILs, and identified six QTLs associated with tassel spindle length in F_2:3_ families and eight QTLs related to tassel spindle length in RILs [26]. However, the genes related to maize tassel spindle length were not identified.

In this study, we selected 182 maize inbred lines with 1,253,814 high-quality SNP markers spanning the entire maize genome. Additionally, a GWAS was conducted to dissect the genetic basis of maize tassel spindle length, with the aim of identifying key candidate genes. 

## 2. Materials and Methods

### 2.1. Experimental Design

The 182 maize inbred lines used in this study were provided by Professor Yan Jianbing’s group at Huazhong Agricultural University. The associated populations were grown in 2023 at the following three locations: Yuanyang Science and Education Park, Henan Agricultural University (YYS; 35°10′66″ N, 113°95′30″ E); Experimental Base of Hebi Academy of Agricultural Sciences (HB; 35°66′60″ N, 114°30′28″ E); and Liaoning Huihe Agricultural Technology Co., Ltd., Tieling County (TL; 42°31′54″ N, 123°60′43″ E). Field experiments were conducted using a completely randomized block design, with each inbred line replicated twice. Plants were grown in single rows (3 m long and separated by 0.65 m) at a density of 67,500 plants ha^−1^. Standard field management practices were followed throughout the field experiments.

### 2.2. Phenotypic Survey

Once the tassels were fully exposed and stabilized in form, the tassel spindle length was examined. Three random plants per row were selected. Specifically, the tassel spindle length was determined by measuring the distance from the lowest branch of the tassel spindle to its top.

### 2.3. Phenotypic Data Analysis and Processing

The phenotypic data were analyzed using Microsoft Excel 2021 and IBM SPSS Statistics 27. The phenotypic data exceeding ± 1.5 times the standard deviation were eliminated and not used for further analysis. To mitigate the effects of environmental variability, correlations among environments (YYS, HB, and TL) were assessed using devtools in R. The best linear unbiased prediction (BLUP) for tassel spindle length across the three environments (YYS, HB, and TL) was determined using lme4 in R (R Core Team). The broad-sense heritability (*h*^2^) of the tassel spindle length was calculated using the following formula: *h*^2^ = δg^2^/(δg^2^ + δe^2^/nr), where δg^2^ represents genetic variance, δe^2^ denotes error variance, n signifies the number of environments, and r indicates the number of replicates. Frequency distribution histograms of the data were generated using Origin 2021.

### 2.4. Genotype and GWAS

The genotype data for the associated populations were downloaded from the website of Prof. Yan Jianbing’s laboratory at Huazhong Agricultural University [27]. The phenotypic data were analyzed using Q, K, and Q + K models and the Tassel 3.0 software. The results were visualized in a QQ plot, a Manhattan plot, and a composite QQ plot using CMplot in R (R Core Team). The best model for controlling false negatives and false positives was selected through the QQ plot. To assess the significance of the association between the phenotype and genotype, *p* ≤ 1.0 × 10^−4^ was set as the threshold [28].

### 2.5. Candidate Gene Identification

The upstream and downstream regions (30 kb) flanking each significant SNP were used as a QTL [29]. Referring to maize inbred line B73 genome V4 (RefGen_V4), the MaizeGDB (https://maizegdb.org/, accessed on 10 September 2024) database was used to find candidate genes within the 30 kb regions, candidate gene functional annotations, and expression patterns. Candidate gene functions in biological pathways were determined according to a KEGG enrichment analysis (https://www.omicshare.com/tools/home/report/koenrich.html, accessed on 18 June 2024). A haplotype analysis was performed using Tassel 3.0, Microsoft Excel 2021, and WPS (12.1.0.18608).

## 3. Results

### 3.1. Phenotypic Analysis of Tassel Spindle Length

The average tassel spindle length across three experimental sites and BLUP ranged from 30.08 to 34.77 cm. In YYS, TL, HB, and BLUP, the maize tassel spindle length ranges were as follows: 19.41–46.50 cm, 19.37–49.57 cm, 16.37–42.50 cm, and 19.98–43.55 cm, respectively (Table 1). The absolute values of skewness and kurtosis for tassel spindle length were less than 1 (Table 1). The frequency distribution histograms of tassel spindle length (Figure S1) revealed the normal distribution of maize tassel spindle length, indicative of a typical quantitative trait. The heritability of maize tassel spindle length was 0.89, suggesting that this trait is primarily controlled by genetic factors but is also influenced by environmental factors (Table 1).

**Figure 1:**
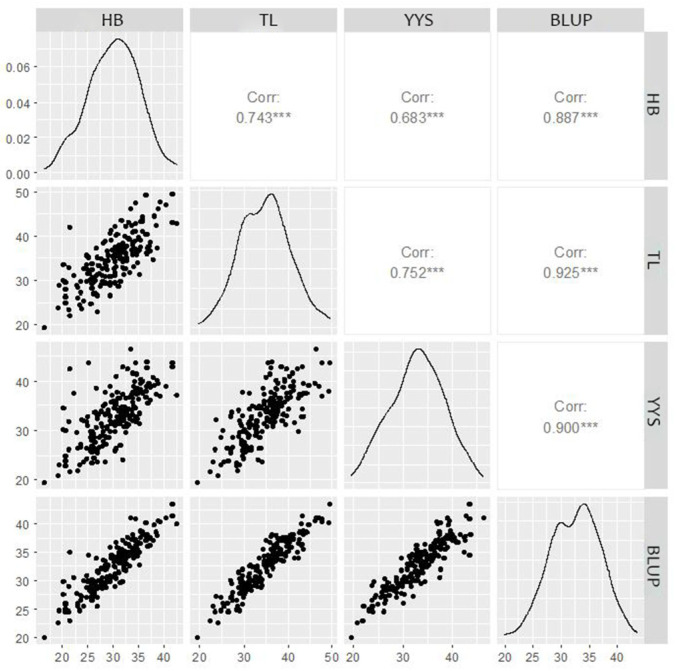
Correlation analysis of maize tassel spindle length. Note: *** Significant correlation at the 0.001 level (both sides); YYS, Yuanyang; TL, Tieling; HB, Hebi; and BLUP, BLUP of Yuanyang, Tieling, and Hebi.

Correlation analysis results reflected the significant positive correlations among tassel spindle length traits across three experimental sites and between each site and BLUP, further highlighting the genetic influence on maize tassel spindle length (Figure 1). Specifically, the correlation coefficients for tassel spindle length were as follows: 0.743 (between HB and TL); 0.683 (between HB and YYS); 0.887 (between HB and BLUP); 0.752 (between YYS and TL); 0.9 (between YYS and BLUP); and 0.925 (between TL and BLUP) (Figure 1).

### 3.2. Genome-Wide Association Analysis

#### 3.2.1. Interpretation of Association Analysis Results

**Figure 2:**
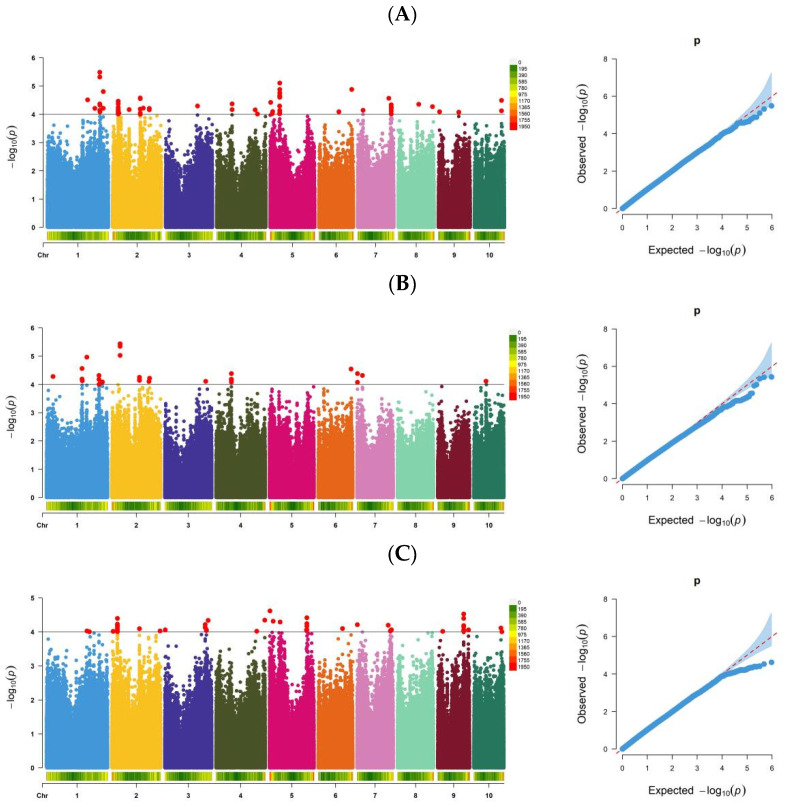

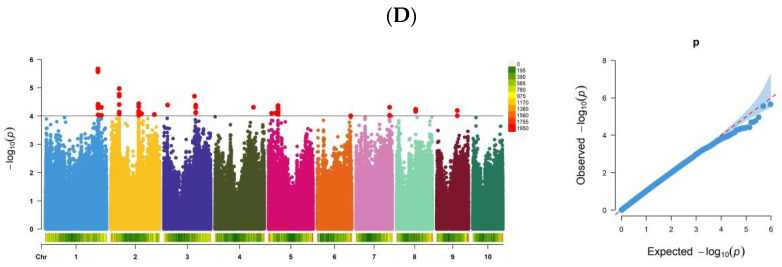
Manhattan plots of maize tassel spindle length. Note: (**A**) BLUP; (**B**) Hebi; (**C**) Tieling; and (**D**) Yuanyang.

Three models (Q, K, and Q + K) were used to analyze tassel spindle length across three experimental sites and BLUP (Figure S2). The composite QQ plot indicated that the Q model had limited control over false positives, whereas the K and Q + K models were better for controlling false negatives and false positives. Considering the widespread use of the Q+K model for GWAS [30,31,32], it was selected for our GWAS. Using the Q+K model and a significance threshold of *p* ≤ 1.0 × 10^−4^, a total of 240 SNPs significantly related to tassel spindle length were identified across three experimental sites and BLUP (Figure 2). These SNPs were associated with 99 QTLs. Specifically, 91 SNP loci significantly linked to tassel spindle length in BLUP were associated with 33 QTLs, explaining 9.83–15.77% of the phenotypic variance; 40 SNP loci related to tassel spindle length at HB were associated with 18 QTLs, explaining 9.64–14.59% of the phenotypic variance; 62 SNP loci related to tassel spindle length at TL were associated with 28 QTLs, explaining 9.60–12.27% of the phenotypic variance; and 47 SNP loci related to tassel spindle length at YYS were associated with 20 QTLs, explaining 10.00–16.35% of the phenotypic variance (Table S1).

#### 3.2.2. Co-Localization Analysis

A simultaneous examination of the data across three experimental sites and BLUP revealed 99 QTLs, 21 of which were detected in two or more environments. Specifically, BLUP and HB shared eight co-localized QTLs (Co-QTLs) associated with 16 genes, explaining 10.20–15.77% of the phenotypic variance; BLUP and TL shared seven Co-QTLs associated with 21 genes, explaining 10.00–13.88% of the phenotypic variance; BLUP and YYS shared 10 Co-QTLs associated with 21 genes, explaining 9.83–16.35% of the phenotypic variance; HB and TL shared one Co-QTL associated with two genes, explaining 10.45–10.51% of the phenotypic variance; and HB and YYS shared three Co-QTLs associated with five genes, explaining 10.58–16.35% of the phenotypic variance. Moreover, BLUP, HB, and TL shared one Co-QTL associated with two genes, explaining 10.20–10.51% of the phenotypic variance, whereas BLUP, HB, and YYS shared three Co-QTLs associated with five genes, explaining 10.58–16.35% of the phenotypic variance (Table 2).

### 3.3. Candidate Gene Analysis

**Figure 3:**
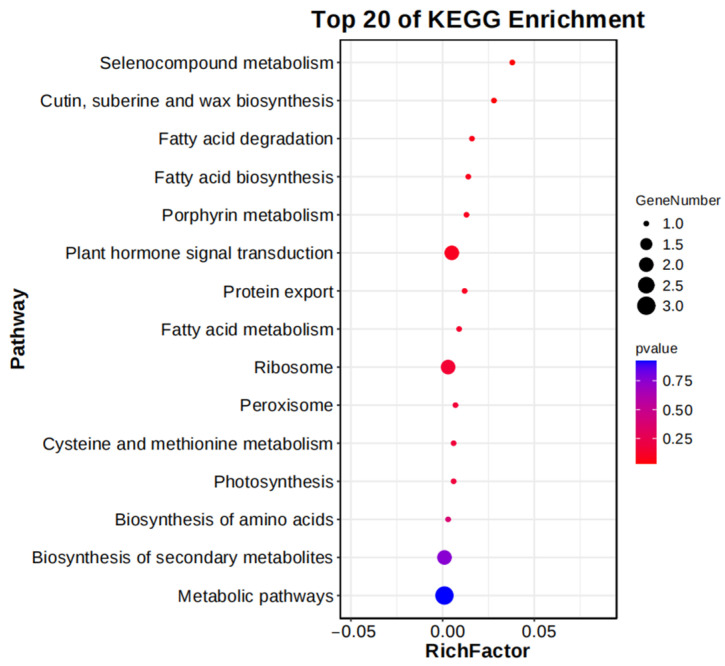
KEGG enrichment analysis of candidate genes affecting maize tassel spindle length.

To identify genes potentially influencing maize tassel spindle length, we conducted a KEGG enrichment analysis of 51 genes associated with 21 Co-QTLs. Significantly enriched KEGG pathways among the candidate genes included “Plant Hormone Signal Transduction” and “Ribosome”. Previous research revealed the regulatory effects of genes related to plant hormone signal transduction on maize tassel spindle length [33], including *Zm00001d031798* and *Zm00001d042312* (Figure 3).

**Figure 4:**
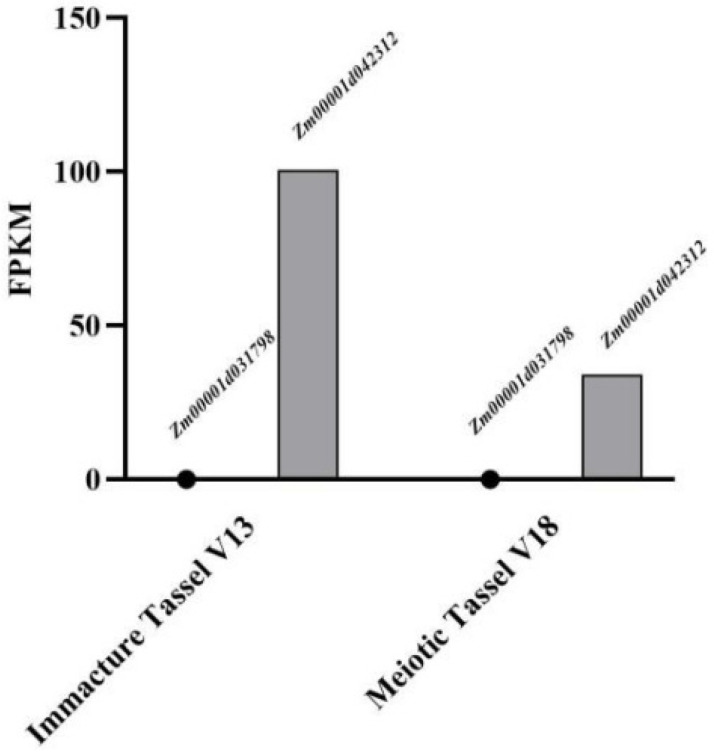
Analysis of the expression of candidate genes affecting maize tassel spindle length.

An analysis of the expression patterns of *Zm00001d042312* and *Zm00001d031798* indicated that *Zm00001d031798* is not expressed in the maize tassel in the V13 and V18 stage. Conversely, *Zm00001d042312* is highly expressed in the maize tassel (Figure 4). Accordingly, *Zm00001d042312* was identified as a candidate gene regulating maize tassel spindle length.

### 3.4. Haplotype Analysis

**Figure 5:**
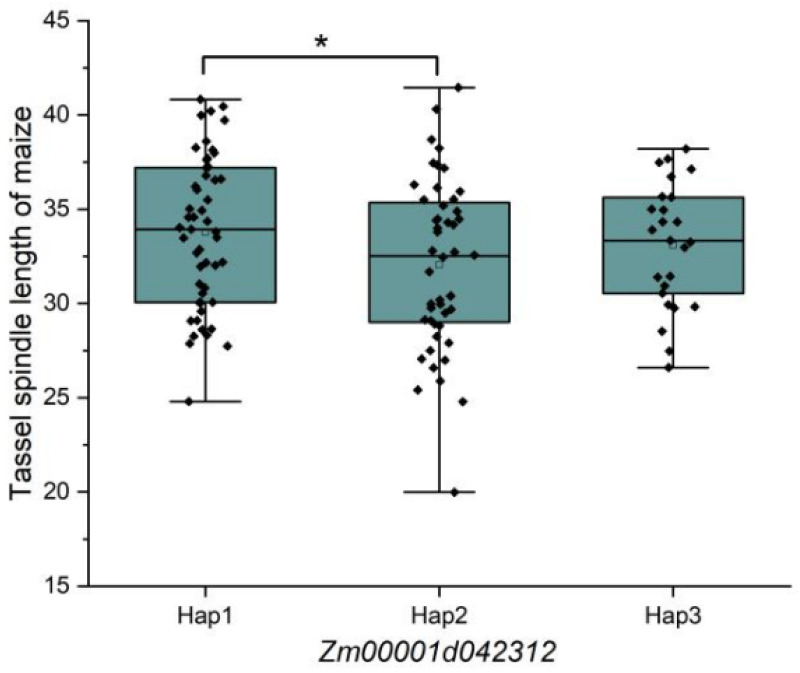
Haplotype analysis of *Zm00001d042312*. Note: * *p* < 0.05.

Based on the BLUP values for maize tassel spindle length, a haplotype analysis was performed for *Zm00001d042312*, which revealed three haplotypes: Hap1 (TTTGTTG), Hap2 (GCCACAT), and Hap3 (GTCGTTG). Among these haplotypes, Hap1, Hap2, and Hap3 were detected in 51, 48, and 25 inbred lines, respectively (Table 3). Significant differences were detected between Hap1 and Hap2 (Figure 5). Thus, *Zm00001d042312* was identified as a key gene influencing maize tassel spindle length.

## 4. Discussion

Earlier research confirmed GWAS involve testing genetic variants across the genomes of many individuals to identify genotype phenotype associations [34]. It has often been used to clarify the genetic basis of important agronomic traits in various crops, including maize. Sun et al. leveraged data for 338 maize inbred lines and multiple omics datasets to elucidate molecular mechanisms regulating maize drought resistance and yield [35]. Yan et al. identified a gene controlling maize grain iron transport on the data for 273 maize inbred lines and six materials exhibiting differential grain iron content [36]. Li et al. conducted a GWAS involving 201 maize inbred lines and detected nine SNPs significantly associated with the maize leaf architecture [37]. A linkage analysis can determine the genetic distance between genes and molecular markers according to the recombination frequency of genetic markers, making it important for gene mapping. Compared with a linkage analysis, GWAS offers several advantages. First, GWAS uses information regarding genetic variations across the entire genome, enabling the higher resolution power for gene mapping. Second, GWAS eliminates the need for constructing complex genetic populations, thereby streamlining the process of gene mapping. Third, GWAS is useful for identifying multiple natural variations [38].

Maize tassel spindle length, which is a key agronomic trait, influences the seed setting rate by modulating pollen quantity and flowering duration. Additionally, an ideal tassel architecture facilitates the flow of nutrients and energy toward the ear, with positive effects on maize yield. Heritability is an important index for the genetic trait’s robustness. In this study, the heritability of the maize tassel spindle length was 0.89. And previous studies indicated that the heritability was 0.683–0.95 [22,23,25,26]. These results further showed that the maize tassel spindle length is a relatively stable genetic trait. In the current study, we focused on 182 maize inbred lines and conducted a GWAS of tassel spindle length using 1,253,814 SNP markers covering the entire maize genome as well as the Q + K model. Our analysis identified 240 SNP loci significantly associated with the tassel spindle length. A total of 21 QTLs were detected in two or more environments. A comparative analysis involving data generated in earlier studies revealed eight previously reported Co-QTLs: Co-QTL5 on chromosome 1; Co-QTL1, Co-QTL6, Co-QTL9, Co-QTL10, and Co-QTL16 on chromosome 2; Co-QTL22 on chromosome 9; and Co-QTL14 on chromosome 10 [20,21]. Additionally, 13 Co-QTLs were not detected in previous studies, possibly because of the differences in materials, study methods, phenotypic plasticity, and environment. These newly identified QTLs associated with tassel spindle length represent novel target loci for breeding superior maize varieties with optimal tassel architecture. In addition, *Zm00001d042312* was putative to the gene required for the maize tassel spindle length. *Zm00001d042312* had three main haplotypes, with significant differences between Hap1 and Hap2. Based on the sequence difference of Hap1 and Hap2, early selection of maize tassel spindle length can be carried out through genetic markers, thereby improving breeding efficiency. The discovery of new QTLs or genes lays the foundation for gene cloning and the dissection of molecular mechanisms underlying the maize tassel spindle length.

Plant hormones are key molecules influencing plant growth and development, with significant roles in diverse processes (e.g., cell division, differentiation, and elongation) [39]. In maize, these hormones have been linked to tassel morphology by regulating inflorescence meristem development. Wang et al. proposed that cytokinin and gibberellin are involved in the inflorescence meristem activity and maintenance, while auxin regulates axillary meristem initiation [40]. In the current study, we completed a GWAS and analyzed candidate genes in terms of their enriched KEGG pathways and expression patterns. Based on the data generated, *Zm00001d042312* was identified as a candidate gene that regulates maize tassel spindle length. This gene encodes a histidine kinase potentially involved in plant hormone signal transduction. In rice, *OHK2*, which is a homolog of *Zm00001d042312*, functions as a cytokinin receptor. The *OHK2* gene encodes a protein with a CHASE domain at the N-terminus, transmembrane domains on both sides of the CHASE domain, a histidine kinase domain in the middle, and a receiver domain at the C-terminus. Hence, *OHK2* may be a cytokinin-signaling-related gene in rice [41]. Choi et al. further identified another histidine kinase, OsHk6, as a cytokinin receptor [42]. In Arabidopsis, *AHK3*, which is homologous to *Zm00001d042312*, encodes a histidine kinase and regulates cytokinin signal transduction [43]. Therefore, we hypothesize that Zm00001d042312, as the cytokinin receptor, is involved in the cytokinin signaling transduction and then mediates the cell division in the male inflorescence meristem, ultimately contributing to the regulation of maize tassel spindle length. Additionally, cytokinin can interact with light signals to regulate plant growth and development, which relies on the red-light receptor *PHYB* and the blue-light receptor *CRY1* [44]. These findings suggest that light spectral quality affects the cytokinin signaling transduction. Hence, we speculate that light spectral quality regulates cytokinin signal transduction by influencing *Zm00001d042312* gene expression, and then modulates the maize tassel spindle length.

## 5. Conclusions

In this study, 182 maize inbred lines were included in an association population. Additionally, a genome-wide association study was conducted on maize tassel spindle length using the Q + K model. With *p* ≤ 1.0 × 10^−4^ applied as the significance threshold, 240 SNPs significantly associated with tassel spindle length were detected, which were associated with 99 QTLs, with 21 QTLs detected in two or more environments. A KEGG enrichment analysis and candidate gene expression analysis indicated that *Zm00001d042312* affects cytokinin signaling transduction and is highly expressed in maize tassel. A haplotype analysis of *Zm00001d042312* revealed three main haplotypes, with significant differences between Hap1 and Hap2. In conclusion, we propose that *Zm00001d042312* is a gene that regulates maize tassel spindle length.

## Figures and Tables

**Table 1:** Statistical analysis of maize tassel spindle length.

Environment	Mean (cm)	Range (cm)	Standard Deviation	Skewness	Kurtosis	Heritability
YYS	32.95	19.41–46.50	5.41	−0.08	−0.41	0.89
TL	34.77	19.37–49.57	5.69	0.10	−0.13	
HB	30.08	16.37–42.50	4.97	−0.14	−0.20	
BLUP	32.60	19.98–43.55	4.30	−0.12	−0.32	

Note: YYS, Yuanyang; TL, Tieling; HB, Hebi; and BLUP, BLUP of Yuanyang, Tieling, and Hebi.

**Table 2:** Co-location of candidate genes and functional annotations of maize tassel spindle length.

Loci	QTL Name	Chr	Environment	Peak (bp)	*p* Value	Contribution Value	Candidate Gene	Annotation
*Co-QTL1*	*QTL-12*	2	BLUP	138477322	9.08 × 10^−5^	10.20%	*Zm00001d004775*	O-fucosyltransferase family protein
							*Zm00001d004778*	Pentatricopeptide repeat-containing protein mitochondrial
	*QTL-43*	2	Hebi	138477322	7.17 × 10^−5^	10.45%	*Zm00001d004775*	O-fucosyltransferase family protein
							*Zm00001d004778*	Pentatricopeptide repeat-containing protein mitochondrial
	*QTL-57*	2	Tieling	138477322	8.05 × 10^−5^	10.51%	*Zm00001d004775*	O-fucosyltransferase family protein
							*Zm00001d004778*	Pentatricopeptide repeat-containing protein mitochondrial
*Co-QTL2*	*QTL-3*	1	BLUP	263536337	3.23 × 10^−6^	15.77%	*Zm00001d033467*	DWNN domain a CCHC-type zinc finger
							*Zm00001d033468*	Lactoylglutathione lyase/glyoxalase I family protein
							*Zm00001d033469*	Ferredoxin
	*QTL-37*	1	Hebi	263536337	4.80 × 10^−5^	12.16%	*Zm00001d033467*	DWNN domain a CCHC-type zinc finger
							*Zm00001d033468*	Lactoylglutathione lyase/glyoxalase I family protein
							*Zm00001d033469*	Ferredoxin
	*QTL-80*	1	Yuanyang	263536337	2.16 × 10^−6^	16.35%	*Zm00001d033467*	DWNN domain a CCHC-type zinc finger
							*Zm00001d033468*	Lactoylglutathione lyase/glyoxalase I family protein
							*Zm00001d033469*	Ferredoxin
*Co-QTL3*	*QTL-4*	1	BLUP	263584988	4.81 × 10^−6^	13.88%	*Zm00001d033470*	Cytochrome P450 family 87 subfamily A polypeptide 2
	*QTL-38*	1	Hebi	263584988	6.71 × 10^−5^	10.58%	*Zm00001d033470*	Cytochrome P450 family 87 subfamily A polypeptide 2
	*QTL-81*	1	Yuanyang	263584988	2.73 × 10^−6^	14.78%	*Zm00001d033470*	Cytochrome P450 family 87 subfamily A polypeptide 2
*Co-QTL4*	*QTL-6*	1	BLUP	280483906	1.58 × 10^−5^	12.92%	*Zm00001d033988*	Cytokinin-O-glucosyltransferase 1
	*QTL-40*	1	Hebi	280483542	8.23 × 10^−5^	10.93%	*Zm00001d033988*	Cytokinin-O-glucosyltransferase 1
	*QTL-83*	1	Yuanyang	280483906	5.00 × 10^−5^	11.10%	*Zm00001d033988*	Cytokinin-O-glucosyltransferase 1
*Co-QTL5*	*QTL-1*	1	BLUP	202184503	3.09 × 10^−5^	12.44%	*Zm00001d031797*	NA
							*Zm00001d031798*	BRI1 kinase inhibitor 1
							*Zm00001d031800*	NA
							*Zm00001d031801*	Putative Carboxylesterase 2
							*Zm00001d031799*	3-β hydroxysteroid dehydrogenase/isomerase
	*QTL-36*	1	Hebi	202184503	1.09 × 10^−5^	14.59%	*Zm00001d031797*	NA
							*Zm00001d031798*	BRI1 kinase inhibitor 1
							*Zm00001d031799*	3-β hydroxysteroid dehydrogenase/isomerase
							*Zm00001d031800*	NA
							*Zm00001d031801*	Putative Carboxylesterase 2
*Co-QTL6*	*QTL-11*	2	BLUP	138312707	2.65 × 10^−5^	11.43%	*Zm00001d004773*	NA
	*QTL-42*	2	Hebi	138312944	5.62 × 10^−5^	10.51%	*Zm00001d004773*	NA
*Co-QTL7*	*QTL-16*	4	BLUP	77406352	4.27 × 10^−5^	11.00%	*Zm00001d050267*	Putative YDG/SRA domain containing protein
							*Zm00001d050268*	NA
	*QTL-48*	4	Hebi	77406352	6.75 × 10^−5^	10.71%	*Zm00001d050267*	Putative YDG/SRA domain containing protein
							*Zm00001d050268*	NA
*Co-QTL8*	*QTL-25*	6	BLUP	164588607	1.31 × 10^−5^	13.21%	*Zm00001d038797*	P-loop nucleoside triphosphate hydrolases superfamily protein with CH (Calponin Homology) domain
	*QTL-49*	6	Hebi	164588607	2.85 × 10^−5^	12.46%	*Zm00001d038797*	P-loop nucleoside triphosphate hydrolases superfamily protein with CH (Calponin Homology) domain
*Co-QTL9*	*QTL-7*	2	BLUP	27832468	8.02 × 10^−5^	10.09%	*Zm00001d002946*	Probable magnesium transporter
							*Zm00001d002945*	NAC transcription factor
	*QTL-55*	2	Tieling	27832468	7.06 × 10^−5^	10.20%	*Zm00001d002946*	Probable magnesium transporter
							*Zm00001d002945*	NAC transcription factor
*Co-QTL10*	*QTL-8*	2	BLUP	27930511	3.45 × 10^−5^	11.18%	*Zm00001d002949*	NA
							*Zm00001d002950*	Protein CHROMATIN REMODELING 24
							*Zm00001d002951*	Protein STAY-GREEN LIKE chloroplastic
	*QTL-56*	2	Tieling	27934487	5.94 × 10^−5^	10.77%	*Zm00001d002949*	NA
							*Zm00001d002950*	Protein CHROMATIN REMODELING 24
							*Zm00001d002951*	Protein STAY-GREEN LIKE chloroplastic
*Co-QTL11*	*QTL-18*	4	BLUP	204772542	9.73 × 10^−5^	10.62%	*Zm00001d052917*	Anaphase-promoting complex subunit 4
							*Zm00001d052918*	Ubiquitin-protein ligase/zinc ion binding protein
	*QTL-63*	4	Tieling	204772542	9.61 × 10^−5^	10.43%	*Zm00001d052917*	Anaphase-promoting complex subunit 4
							*Zm00001d052918*	Ubiquitin-protein ligase/zinc ion binding protein
*Co-QTL12*	*QTL-19*	5	BLUP	2894790	3.81 × 10^−5^	10.78%	*Zm00001d012992*	40S ribosomal protein S26
							*Zm00001d012993*	Transcription elongation factor
							*Zm00001d012994*	Histidine kinase
							*Zm00001d012996*	NA
							*Zm00001d012998*	50S ribosomal protein L17
							*Zm00001d012991*	2-oxoglutarate-dependent dioxygenase family protein
							*Zm00001d012995*	NA
	*QTL-65*	5	Tieling	2894790	2.42 × 10^−5^	11.33%	*Zm00001d012991*	2-oxoglutarate-dependent dioxygenase family protein
							*Zm00001d012992*	40S ribosomal protein S26
							*Zm00001d012993*	Transcription elongation factor
							*Zm00001d012994*	Histidine kinase
							*Zm00001d012995*	NA
							*Zm00001d012996*	NA
							*Zm00001d012998*	50S ribosomal protein L17
*Co-QTL13*	*QTL-26*	7	BLUP	156487883	2.72 × 10^−5^	13.88%	*Zm00001d021573*	SBP transcription factor 29
							*Zm00001d021574*	MAPK activating protein
	*QTL-72*	7	Tieling	156487883	6.37 × 10^−5^	12.27%	*Zm00001d021573*	SBP transcription factor 29
							*Zm00001d021574*	MAPK activating protein
*Co-QTL14*	*QTL-33*	10	BLUP	135687334	3.21 × 10^−5^	11.10%	*Zm00001d025996*	Ubiquitin carboxyl-terminal hydrolase family protein
							*Zm00001d025997*	Non-specific serine/threonine protein kinase
							*Zm00001d025998*	Ubiquitin carboxyl-terminal hydrolase
	*QTL-78*	10	Tieling	135687334	7.69 × 10^−5^	10.00%	*Zm00001d025996*	Ubiquitin carboxyl-terminal hydrolase family protein
							*Zm00001d025997*	Non-specific serine/threonine protein kinase
							*Zm00001d025998*	Ubiquitin carboxyl-terminal hydrolase
*Co-QTL15*	*QTL-5*	1	BLUP	263887558	4.77 × 10^−5^	10.49%	*Zm00001d033478*	HSP20-like chaperones superfamily protein
							*Zm00001d033479*	Signal peptidase complex subunit 1
							*Zm00001d033480*	5-methyltetrahydropteroyltriglutamate--homocysteine methyltransferase
							*Zm00001d033481*	Protein FAR1-RELATED SEQUENCE 5
	*QTL-82*	1	Yuanyang	263887558	3.84 × 10^−5^	10.67%	*Zm00001d033478*	HSP20-like chaperones superfamily protein
							*Zm00001d033479*	Signal peptidase complex subunit 1
							*Zm00001d033480*	5-methyltetrahydropteroyltriglutamate--homocysteine methyltransferase
							*Zm00001d033481*	Protein FAR1-RELATED SEQUENCE 5
*Co-QTL16*	*QTL-13*	2	BLUP	154142006	6.06 × 10^−5^	10.23%	*Zm00001d005019*	4-coumarate—CoA ligase
	*QTL-86*	2	Yuanyang	154142006	8.17 × 10^−5^	10.00%	*Zm00001d005019*	4-coumarate—CoA ligase
*Co-QTL17*	*QTL-15*	3	BLUP	160486456	5.07 × 10^−5^	10.78%	*Zm00001d042312*	Histidine kinase2
							*Zm00001d042313*	Calmodulin-binding transcription activator 4
	*QTL-90*	3	Yuanyang	160487429	4.14 × 10^−5^	12.33%	*Zm00001d042312*	Histidine kinase2
							*Zm00001d042313*	Calmodulin-binding transcription activator 4
*Co-QTL18*	*QTL-17*	4	BLUP	193040531	6.97 × 10^−5^	12.16%	*Zm00001d052557*	NA
							*Zm00001d052558*	Dirigent protein
	*QTL-92*	4	Yuanyang	193040531	4.95 × 10^−5^	12.77%	*Zm00001d052557*	NA
							*Zm00001d052558*	Dirigent protein
*Co-QTL19*	*QTL-23*	5	BLUP	48260848	7.86 × 10^−6^	12.81%	*Zm00001d014458*	Glutamate receptor
							*Zm00001d014459*	Cytochrome P450 superfamily protein
	*QTL-95*	5	Yuanyang	48260848	4.27 × 10^−5^	10.53%	*Zm00001d014458*	Glutamate receptor 3.4
							*Zm00001d014459*	Cytochrome P450 superfamily protein
*Co-QTL20*	*QTL-27*	7	BLUP	168606046	5.49 × 10^−5^	10.75%	*Zm00001d022045*	tRNA/rRNA methyltransferase (SpoU) family protein
	*QTL-97*	7	Yuanyang	168609483	4.94 × 10^−5^	10.60%	*Zm00001d022045*	tRNA/rRNA methyltransferase (SpoU) family protein
*Co-QTL21*	*QTL-32*	9	BLUP	102108330	8.42 × 10^−5^	10.84%	*Zm00001d046678*	NA
							*Zm00001d046679*	Potassium transporter
							*Zm00001d046680*	Pentatricopeptide repeat-containing protein
							*Zm00001d046681*	Probable glucomannan 4-β-mannosyltransferase 9
	*QTL-99*	9	Yuanyang	102108865	6.37 × 10^−5^	10.22%	*Zm00001d046678*	NA
							*Zm00001d046679*	Potassium transporter
							*Zm00001d046680*	Pentatricopeptide repeat-containing protein
							*Zm00001d046681*	Probable glucomannan 4-β-mannosyltransferase 9

Notes: All QTLs with overlapping QTL regions were categorized as a locus; physical position of each SNP was based on B73 RefGen_V4; *p* value of the corresponding trait was calculated by Q + K model; and the phenotypic variance was explained by the corresponding locus.

**Table 3:** Haplotype analysis of *Zm00001d042312*.

	SNP160483225	SNP160476977	SNP160475587	SNP160477994	SNP160477898	SNP160482362	SNP160481029
Hap1	T	T	T	G	T	T	G
Hap2	G	C	C	A	C	A	T
Hap3	G	T	C	G	T	T	G

Note: Hap1, Hap2, and Hap3 were detected in 51, 48, and 25 inbred lines, respectively.

## Data Availability

The datasets supporting the conclusions of this article are included. within the article (and its supplementary files). Genotypic data can be downloaded from http://www.maizego.org/Resources.html.

## Supplementary Materials

The following supporting information can be downloaded at: https://www.mdpi.com/article/10.3390/genes15111413/s1, Figure S1. Frequency distribution of maize tassel spindle length. Figure S2. QQ plots of the GWAS using Q, K, and Q + K models for analyzing maize tassel spindle length. Table S1. Candidate genes and functional annotations of maize tassel spindle length.
