# Supplementary material for: Mining Candidate Genes for Maize Tassel Spindle Length Based on a Genome-Wide Association Analysis
Source: Genes (Basel). 2024 Oct 31;15(11):1413. doi: 10.3390/genes15111413 (PMC11593375; doi:10.3390/genes15111413)
Supplement: Supplementary file 1 [file genes-15-01413-s001.zip › Figure S1. Frequency distribution of maize tassel spindle length.pdf]

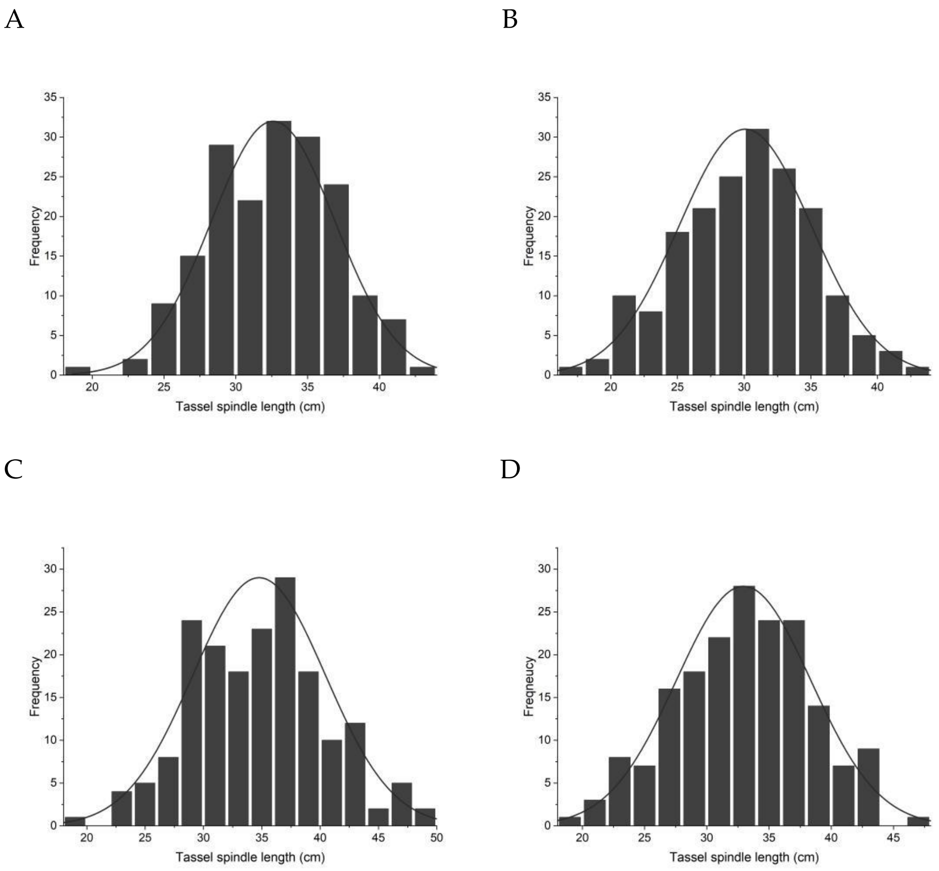

Figure S1. Frequency distribution of maize tassel spindle length.

Note: A: maize tassel spindle length in BLUP; B: maize tassel spindle length in Hebi; C: maize tassel spindle length in Tieling; D: maize tassel spindle length in Yuanyang.
