# Supplementary material for: Mining Candidate Genes for Maize Tassel Spindle Length Based on a Genome-Wide Association Analysis
Source: Genes (Basel). 2024 Oct 31;15(11):1413. doi: 10.3390/genes15111413 (PMC11593375; doi:10.3390/genes15111413)
Supplement: Supplementary file 1 [file genes-15-01413-s001.zip › Figure S2. QQ plots of the GWAS using Q, K, and Q+K models for analyzing maize tassel spindle length..pdf]

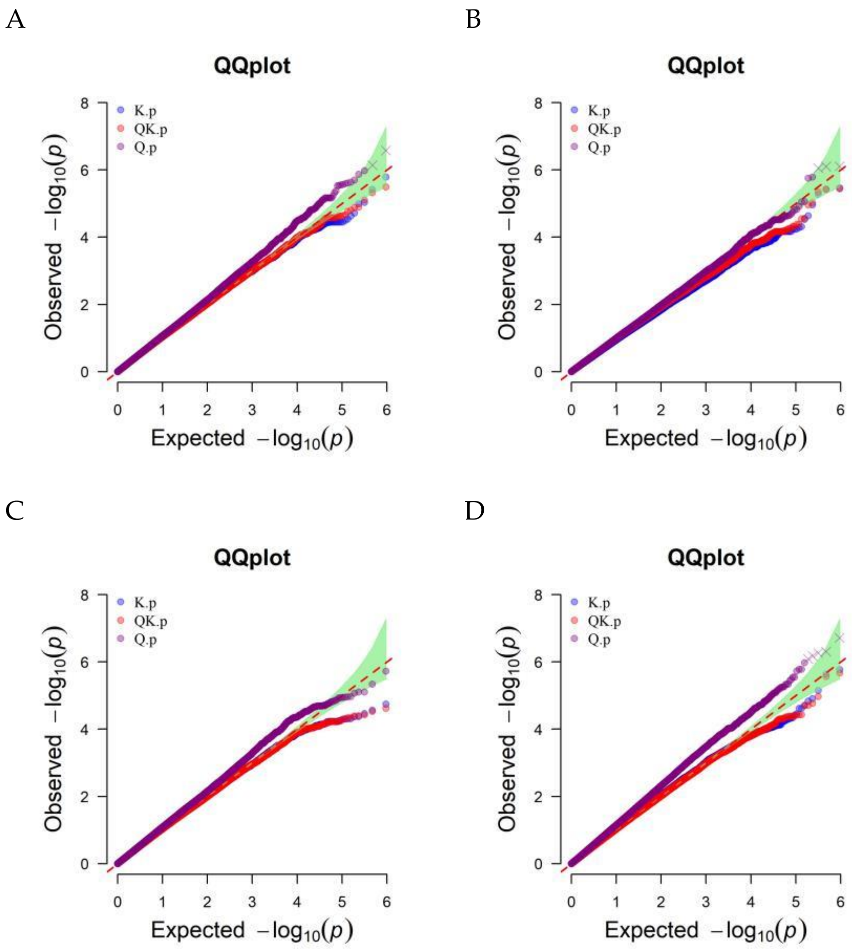

Figure S2. QQ plots of the GWAS using Q, K and Q+K models for analyzing maize tassel spindle length. Note: A, BLUP; B, Hebi; C, Tieling; D, Yuanyang.
