# Supplementary material for: Mining Candidate Genes for Maize Tassel Spindle Length Based on a Genome-Wide Association Analysis
Source: Genes (Basel). 2024 Oct 31;15(11):1413. doi: 10.3390/genes15111413 (PMC11593375; doi:10.3390/genes15111413)
Supplement: Supplementary file 1 [file genes-15-01413-s001.zip › Table S1. Candidate genes and functional annotations of maize tassel spindle length(2).pdf]

**Table S1.** Candidate genes and functional annotations of maize tassel spindle length.

| Loci | QTL name | Chr | Environment | Peak (bp) | P value | Contribution value | Candidate gene | Annotation |
|---|---|---|---|---|---|---|---|---|
| 1 | QTL-1 | 1 | BLUP | 202184503 | 3.09E-05 | 12.44% | *Zm00001d031797* | NA |
| | | | | | | | *Zm00001d031798* | BRI1 kinase inhibitor 1 |
| | | | | | | | *Zm00001d031800* | NA |
| | | | | | | | *Zm00001d031801* | Putative Carboxylesterase 2 |
| | | | | | | | *Zm00001d031799* | 3-beta hydroxysteroid dehydrogenase / isomerase |
| 2 | QTL-2 | 1 | BLUP | 238422595 | 6.20E-05 | 10.16% | *Zm00001d032797* | Probable xyloglucan galactosyltransferase GT15 |
| | | | | | | | *Zm00001d032798* | RNase H family protein |
| 3 | QTL-3 | 1 | BLUP | 263536337 | 3.23E-06 | 15.77% | *Zm00001d033467* | DWNN domain a CCHC-type zinc finger |
| | | | | | | | *Zm00001d033468* | Lactoylglutathione lyase / glyoxalase I family protein |
| | | | | | | | *Zm00001d033469* | Ferredoxin |
| 4 | QTL-4 | 1 | BLUP | 263584988 | 4.81E-06 | 13.88% | *Zm00001d033470* | Cytochrome P450 family 87 subfamily A polypeptide 2 |
| 5 | QTL-5 | 1 | BLUP | 263887558 | 4.77E-05 | 10.49% | *Zm00001d033478* | HSP20-like chaperones superfamily protein |
| | | | | | | | *Zm00001d033479* | Signal peptidase complex subunit 1 |
| | | | | | | | *Zm00001d033480* | 5-methyltetrahydropteroyltriglutamate--homocysteine methyltransferase |
| | | | | | | | *Zm00001d033481* | Protein FAR1-RELATED SEQUENCE 5 |
| 6 | QTL-6 | 1 | BLUP | 280483906 | 1.58E-05 | 12.92% | *Zm00001d033988* | Cytokinin-O-glucosyltransferase 1 |
| 7 | QTL-7 | 2 | BLUP | 27832468 | 8.02E-05 | 10.09% | *Zm00001d002946* | Probable magnesium transporter |
| | | | | | | | *Zm00001d002945* | NAC transcription factor |
| 8 | QTL-8 | 2 | BLUP | 27930511 | 3.45E-05 | 11.18% | *Zm00001d002949* | NA |
| | | | | | | | *Zm00001d002950* | Protein CHROMATIN REMODELING 24 |
| | | | | | | | *Zm00001d002951* | Protein STAY-GREEN LIKE chloroplastic |
| 9 | QTL-9 | 2 | BLUP | 31359270 | 9.01E-05 | 10.54% | *Zm00001d003079* | Protein GPR107 |
| | | | | | | | *Zm00001d003080* | NAC transcription factor |
| 10 | QTL-10 | 2 | BLUP | 82666844 | 6.80E-05 | 11.02% | *Zm00001d004096* | DNA-directed RNA polymerases II IV and V subunit 9A |
| | | | | | | | *Zm00001d004097* | Pleiotropic drug resistance 12 isoform 3 |
| | | | | | | | *Zm00001d004098* | NA |
| 11 | QTL-11 | 2 | BLUP | 138312707 | 2.65E-05 | 11.43% | *Zm00001d004773* | NA |
| 12 | QTL-12 | 2 | BLUP | 138477322 | 9.08E-05 | 10.20% | *Zm00001d004775* | O-fucosyltransferase family protein |
| | | | | | | | *Zm00001d004778* | Pentatricopeptide repeat-containing protein mitochondrial |
| 13 | QTL-13 | 2 | BLUP | 154142006 | 6.06E-05 | 10.23% | *Zm00001d005019* | 4-coumarate--CoA ligase |

| | | | | | | | | |
|---|---|---|---|---|---|---|---|---|
| 14 | QTL-14 | 2 | BLUP | 184174013 | 6.17E-05 | 10.17% | *Zm00001d005711* | Malonyl-CoA decarboxylase family protein |
| 15 | QTL-15 | 3 | BLUP | 160486456 | 5.07E-05 | 10.78% | *Zm00001d042312* | Histidine kinase2 |
| | | | | | | | *Zm00001d042313* | Calmodulin-binding transcription activator 4 |
| 16 | QTL-16 | 4 | BLUP | 77406352 | 4.27E-05 | 11.00% | *Zm00001d050267* | Putative YDG / SRA domain containing protein |
| | | | | | | | *Zm00001d050268* | NA |
| 17 | QTL-17 | 4 | BLUP | 193040531 | 6.97E-05 | 12.16% | *Zm00001d052557* | NA |
| | | | | | | | *Zm00001d052558* | Dirigent protein |
| 18 | QTL-18 | 4 | BLUP | 204772542 | 9.73E-05 | 10.62% | *Zm00001d052917* | Anaphase-promoting complex subunit 4 |
| | | | | | | | *Zm00001d052918* | Ubiquitin-protein ligase/ zinc ion binding protein |
| 19 | QTL-19 | 5 | BLUP | 2894790 | 3.81E-05 | 10.78% | *Zm00001d012992* | 40S ribosomal protein S26 |
| | | | | | | | *Zm00001d012993* | Transcription elongation factor |
| | | | | | | | *Zm00001d012994* | Histidine kinase |
| | | | | | | | *Zm00001d012996* | NA |
| | | | | | | | *Zm00001d012998* | 50S ribosomal protein L17 |
| | | | | | | | *Zm00001d012991* | 2-oxoglutarate-dependent dioxygenase family protein |
| | | | | | | | *Zm00001d012995* | NA |
| 20 | QTL-20 | 5 | BLUP | 10499907 | 9.61E-05 | 9.98% | *Zm00001d013402* | Phytochrome A |
| | | | | | | | *Zm00001d013405* | Protein kinase superfamily protein |
| | | | | | | | *Zm00001d013406* | Alpha/beta-Hydrolases superfamily protein |
| | | | | | | | *Zm00001d013407* | NA |
| 21 | QTL-21 | 5 | BLUP | 12699942 | 8.00E-05 | 9.84% | *Zm00001d013490* | Ubiquitin-like protease family profile domain-containing protein |
| | | | | | | | *Zm00001d013491* | Serine/threonine-protein kinase RUNKEL |
| | | | | | | | *Zm00001d013493* | Lipoxygenase |
| | | | | | | | *Zm00001d013494* | Oleosin |
| | | | | | | | *Zm00001d013492* | Ethylene-insensitive protein 2 |
| 22 | QTL-22 | 5 | BLUP | 48034403 | 2.26E-05 | 11.45% | *Zm00001d014455* | Remorin family protein |
| | | | | | | | *Zm00001d014456* | Glutamate receptor |
| 23 | QTL-23 | 5 | BLUP | 48260848 | 7.86E-06 | 12.81% | *Zm00001d014458* | Glutamate receptor |
| | | | | | | | *Zm00001d014459* | Cytochrome P450 superfamily protein |
| 24 | QTL-24 | 6 | BLUP | 100073657 | 8.15E-05 | 10.38% | *Zm00001d036763* | Putative cytochrome P450 superfamily protein |
| | | | | | | | *Zm00001d036764* | Putative methyltransferase family protein |
| | | | | | | | *Zm00001d036765* | RING-type E3 ubiquitin transferase |
| 25 | QTL-25 | 6 | BLUP | 164588607 | 1.31E-05 | 13.21% | *Zm00001d038797* | P-loop nucleoside triphosphate hydrolases superfamily protein with CH |

| | | | | | | | | |
|---|---|---|---|---|---|---|---|---|
| | | | | | | | | (Calponin Homology) domain |
| 26 | QTL-26 | 7 | BLUP | 156487883 | 2.72E-05 | 13.88% | *Zm00001d021573* | SBP transcription factor 29 |
| | | | | | | | *Zm00001d021574* | MAPK activating protein |
| 27 | QTL-27 | 7 | BLUP | 168606046 | 5.49E-05 | 10.75% | *Zm00001d022045* | tRNA/rRNA methyltransferase (SpoU) family protein |
| 28 | QTL-28 | 7 | BLUP | 169044172 | 5.86E-05 | 10.37% | *Zm00001d022050* | RNA-binding (RRM/RBD/RNP motifs) family protein |
| | | | | | | | *Zm00001d022051* | Peptidase M1 family protein |
| | | | | | | | *Zm00001d022052* | Putative pentatricopeptide repeat-containing protein |
| 29 | QTL-29 | 8 | BLUP | 103221937 | 4.44E-05 | 10.64% | *Zm00001d010178* | Protein kinase superfamily protein |
| 30 | QTL-30 | 8 | BLUP | 172717088 | 5.36E-05 | 13.53% | *Zm00001d012336* | Calcium-dependent lipid-binding (CaLB domain) family protein |
| | | | | | | | *Zm00001d012337* | MYB-type transcription factor |
| | | | | | | | *Zm00001d012338* | Coronatine-insensitive protein 1 |
| | | | | | | | *Zm00001d012339* | NA |
| | | | | | | | *Zm00001d012340* | Pentatricopeptide repeat-containing protein |
| | | | | | | | *Zm00001d012341* | Guanylyl cyclase 1 |
| | | | | | | | *Zm00001d012342* | Guanine nucleotide exchange factor SPIKE 1 |
| 31 | QTL-31 | 9 | BLUP | 5654116 | 8.15E-05 | 10.68% | *Zm00001d044860* | TLD-domain containing nucleolar protein |
| | | | | | | | *Zm00001d044861* | NA |
| 32 | QTL-32 | 9 | BLUP | 102108330 | 8.42E-05 | 10.84% | *Zm00001d046678* | NA |
| | | | | | | | *Zm00001d046679* | Potassium transporter |
| | | | | | | | *Zm00001d046680* | Pentatricopeptide repeat-containing protein |
| | | | | | | | *Zm00001d046681* | Probable glucomannan 4-beta-mannosyltransferase 9 |
| 33 | QTL-33 | 10 | BLUP | 135687334 | 3.21E-05 | 11.10% | *Zm00001d025996* | Ubiquitin carboxyl-terminal hydrolase family protein |
| | | | | | | | *Zm00001d025997* | Non-specific serine/threonine protein kinase |
| | | | | | | | *Zm00001d025998* | Ubiquitin carboxyl-terminal hydrolase |
| 34 | QTL-34 | 1 | Hebi | 33149700 | 5.28E-05 | 11.97% | *Zm00001d028392* | Actin depolymerizing factor5 |
| | | | | | | | *Zm00001d028393* | UDP-N-acetylglucosamine diphosphorylase 2 |
| | | | | | | | *Zm00001d028394* | Bifunctional D-cysteine desulfhydrase /1-aminocyclopropane-1-carboxylate deaminase mitochondrial |
| | | | | | | | *Zm00001d028395* | Fructose-bisphosphate aldolase |
| 35 | QTL-35 | 1 | Hebi | 178043365 | 6.48E-05 | 11.66% | *Zm00001d031120* | Cationic amino acid transporter 4 vacuolar |
| 36 | QTL-36 | 1 | Hebi | 202184503 | 1.09E-05 | 14.59% | *Zm00001d031797* | NA |
| | | | | | | | *Zm00001d031798* | BRI1 kinase inhibitor 1 |
| | | | | | | | *Zm00001d031799* | 3-beta hydroxysteroid dehydrogenase / isomerase |

| | | | | | | | | |
|---|---|---|---|---|---|---|---|---|
| | | | | | | | *Zm00001d031800* | NA |
| | | | | | | | *Zm00001d031801* | Putative Carboxylesterase 2 |
| 37 | QTL-37 | 1 | Hebi | 263536337 | 4.80E-05 | 12.16% | *Zm00001d033467* | DWNN domain a CCHC-type zinc finger |
| | | | | | | | *Zm00001d033468* | Lactoylglutathione lyase / glyoxalase I family protein |
| | | | | | | | *Zm00001d033469* | Ferredoxin |
| 38 | QTL-38 | 1 | Hebi | 263584988 | 6.71E-05 | 10.58% | *Zm00001d033470* | Cytochrome P450 family 87 subfamily A polypeptide 2 |
| 39 | QTL-39 | 1 | Hebi | 264211417 | 9.92E-05 | 9.64% | *Zm00001d033492* | High affinity cationic amino acid transporter 1 |
| | | | | | | | *Zm00001d033493* | Cationic amino acid transporter 4 vacuolar |
| 40 | QTL-40 | 1 | Hebi | 280483542 | 8.23E-05 | 10.93% | *Zm00001d033988* | Cytokinin-O-glucosyltransferase 1 |
| 41 | QTL-41 | 2 | Hebi | 41020751 | 3.64E-06 | 14.33% | *Zm00001d003344* | NA |
| | | | | | | | *Zm00001d003345* | Small nuclear ribonucleoprotein Sm D1 |
| | | | | | | | *Zm00001d003346* | NA |
| | | | | | | | *Zm00001d003347* | Ras related protein1 |
| | | | | | | | *Zm00001d003349* | Cytochrome P450 family 724 subfamily A polypeptide 1 |
| 42 | QTL-42 | 2 | Hebi | 138312944 | 5.62E-05 | 10.51% | *Zm00001d004773* | NA |
| 43 | QTL-43 | 2 | Hebi | 138477322 | 7.17E-05 | 10.45% | *Zm00001d004775* | O-fucosyltransferase family protein |
| | | | | | | | *Zm00001d004778* | Pentatricopeptide repeat-containing protein mitochondrial |
| 44 | QTL-44 | 2 | Hebi | 186199371 | 7.85E-05 | 11.03% | *Zm00001d005751* | Golgi SNAP receptor complex member 1 |
| | | | | | | | *Zm00001d005752* | DUF868 family protein |
| | | | | | | | *Zm00001d005753* | 60S ribosomal protein L26-1 |
| 45 | QTL-45 | 2 | Hebi | 189466360 | 6.08E-05 | 10.26% | *Zm00001d005808* | Ethanolamine kinase |
| | | | | | | | *Zm00001d005811* | NA |
| | | | | | | | *Zm00001d005812* | Sterile alpha motif (SAM) domain-containing protein |
| 46 | QTL-46 | 3 | Hebi | 205197675 | 7.76E-05 | 10.48% | *Zm00001d043613* | CHY-type/CTCHY-type/RING-type Zinc finger protein |
| 47 | QTL-47 | 4 | Hebi | 77233111 | 4.13E-05 | 11.01% | *Zm00001d050265* | Hypersensitive-induced response protein 2 |
| 48 | QTL-48 | 4 | Hebi | 77406352 | 6.75E-05 | 10.71% | *Zm00001d050267* | Putative YDG / SRA domain containing protein |
| | | | | | | | *Zm00001d050268* | NA |
| 49 | QTL-49 | 6 | Hebi | 164588607 | 2.85E-05 | 12.46% | *Zm00001d038797* | P-loop nucleoside triphosphate hydrolases superfamily protein with CH (Calponin Homology) domain |
| 50 | QTL-50 | 7 | Hebi | 2162117 | 4.13E-05 | 12.62% | *Zm00001d018667* | MADS3 |
| 51 | QTL-51 | 10 | Hebi | 60725369 | 7.73E-05 | 11.04% | *Zm00001d024272* | Pentatricopeptide repeat-containing protein mitochondrial |
| 52 | QTL-52 | 1 | Tieling | 202993722 | 9.40E-05 | 9.77% | *Zm00001d031822* | Mucin-2 |
| | | | | | | | *Zm00001d031823* | NA |

| | | | | | | | | |
|---|---|---|---|---|---|---|---|---|
| | | | | | | | *Zm00001d031824* | Ribosomal protein S5 domain 2-like superfamily protein |
| | | | | | | | *Zm00001d031825* | ATP synthase subunit d mitochondrial |
| | | | | | | | *Zm00001d031826* | AT-hook motif nuclear-localized protein 17 |
| | | | | | | | *Zm00001d031827* | Ubiquitin-like protease family profile domain-containing protein |
| 53 | QTL-53 | 1 | Tieling | 215245476 | 9.96E-05 | 9.60% | *Zm00001d032164* | DUF868 family protein |
| | | | | | | | *Zm00001d032165* | Defective in cullin neddylation protein |
| 54 | QTL-54 | 2 | Tieling | 6421123 | 9.63E-05 | 9.89% | *Zm00001d002128* | Shikimate kinase 1 chloroplastic |
| | | | | | | | *Zm00001d002129* | Protein kinase superfamily protein |
| | | | | | | | *Zm00001d002130* | NA |
| | | | | | | | *Zm00001d002131* | 2-Sep |
| | | | | | | | *Zm00001d002132* | NA |
| | | | | | | | *Zm00001d002133* | NA |
| | | | | | | | *Zm00001d002134* | NA |
| | | | | | | | *Zm00001d002135* | FGGY family of carbohydrate kinase |
| 55 | QTL-55 | 2 | Tieling | 27832468 | 7.06E-05 | 10.20% | *Zm00001d002946* | Probable magnesium transporter |
| | | | | | | | *Zm00001d002945* | NAC transcription factor |
| 56 | QTL-56 | 2 | Tieling | 27934487 | 5.94E-05 | 10.77% | *Zm00001d002949* | NA |
| | | | | | | | *Zm00001d002950* | Protein CHROMATIN REMODELING 24 |
| | | | | | | | *Zm00001d002951* | Protein STAY-GREEN LIKE chloroplastic |
| 57 | QTL-57 | 2 | Tieling | 138477322 | 8.05E-05 | 10.51% | *Zm00001d004775* | O-fucosyltransferase family protein |
| | | | | | | | *Zm00001d004778* | Pentatricopeptide repeat-containing protein mitochondrial |
| 58 | QTL-58 | 2 | Tieling | 241667999 | 9.38E-05 | 9.68% | *Zm00001d007881* | TatD related DNase |
| | | | | | | | *Zm00001d007882* | Nuclear transcription factor Y subunit |
| | | | | | | | *Zm00001d007883* | Glutathione S-transferase T3 |
| | | | | | | | *Zm00001d007884* | MD-2-related lipid recognition domain-containing protein / ML domain-containing protein |
| | | | | | | | *Zm00001d007885* | Glutaredoxin family protein |
| | | | | | | | *Zm00001d007886* | Protein ENHANCED DOWNY MILDEW 2 |
| 59 | QTL-59 | 3 | Tieling | 2014353 | 8.78E-05 | 9.86% | *Zm00001d039330* | V-type proton ATPase subunit E3 |
| | | | | | | | *Zm00001d039331* | V-type proton ATPase subunit E |
| | | | | | | | *Zm00001d039334* | Secreted protein |
| | | | | | | | *Zm00001d039335* | Secreted protein |
| | | | | | | | *Zm00001d039336* | E3 ubiquitin-protein ligase RHF2A |

| | | | | | | | | |
|---|---|---|---|---|---|---|---|---|
| 60 | QTL-60 | 3 | Tieling | 202507497 | 6.06E-05 | 10.28% | *Zm00001d043516* | LysM domain receptor-like kinase 3 |
| | | | | | | | *Zm00001d043517* | Peptidase M28 family protein |
| 61 | QTL-61 | 3 | Tieling | 208734748 | 9.13E-05 | 10.14% | *Zm00001d043737* | AWPM-19-like family protein |
| 62 | QTL-62 | 3 | Tieling | 217463689 | 4.61E-05 | 10.88% | *Zm00001d044037* | Protein trichome birefringence-like 39 |
| | | | | | | | *Zm00001d044038* | Tetratricopeptide repeat (TPR)-like superfamily protein |
| | | | | | | | *Zm00001d044039* | Wax synthase isoform 3 |
| | | | | | | | *Zm00001d044040* | Alpha-L-fucosidase 2 |
| 63 | QTL-63 | 4 | Tieling | 204772542 | 9.61E-05 | 10.43% | *Zm00001d052917* | Anaphase-promoting complex subunit 4 |
| | | | | | | | *Zm00001d052918* | Ubiquitin-protein ligase/ zinc ion binding protein |
| 64 | QTL-64 | 4 | Tieling | 244765103 | 4.51E-05 | 10.88% | *Zm00001d053989* | RING/U-box protein |
| | | | | | | | *Zm00001d053991* | NA |
| | | | | | | | *Zm00001d053993* | NA |
| 65 | QTL-65 | 5 | Tieling | 2894790 | 2.42E-05 | 11.33% | *Zm00001d012991* | 2-oxoglutarate-dependent dioxygenase family protein |
| | | | | | | | *Zm00001d012992* | 40S ribosomal protein S26 |
| | | | | | | | *Zm00001d012993* | Transcription elongation factor |
| | | | | | | | *Zm00001d012994* | Histidine kinase |
| | | | | | | | *Zm00001d012995* | NA |
| | | | | | | | *Zm00001d012996* | NA |
| | | | | | | | *Zm00001d012998* | 50S ribosomal protein L17 |
| 66 | QTL-66 | 5 | Tieling | 19172925 | 4.85E-05 | 10.52% | *Zm00001d013746* | NA |
| | | | | | | | *Zm00001d013747* | Presenilin |
| 67 | QTL-67 | 5 | Tieling | 53683265 | 5.14E-05 | 10.45% | *Zm00001d014568* | DNA repair protein REV1 |
| 68 | QTL-68 | 5 | Tieling | 185771127 | 8.86E-05 | 9.83% | *Zm00001d017110* | Steroid nuclear receptor ligand-binding |
| | | | | | | | *Zm00001d017111* | B-keto acyl reductase |
| | | | | | | | *Zm00001d017112* | B3 domain-containing protein |
| | | | | | | | *Zm00001d017113* | NA |
| | | | | | | | *Zm00001d017114* | Embryo defective 2423 |
| 69 | QTL-69 | 5 | Tieling | 187859720 | 3.83E-05 | 11.34% | *Zm00001d017171* | Outer envelope pore protein 37 chloroplastic |
| 70 | QTL-70 | 6 | Tieling | 122161312 | 7.99E-05 | 10.40% | *Zm00001d037343* | ABA-induced protein |
| 71 | QTL-71 | 7 | Tieling | 1880075 | 6.15E-05 | 10.91% | *Zm00001d018652* | Flavin-containing monooxygenase |
| | | | | | | | *Zm00001d018653* | Purple acid phosphatase |
| 72 | QTL-72 | 7 | Tieling | 156487883 | 6.37E-05 | 12.27% | *Zm00001d021573* | SBP transcription factor 29 |

| | | | | | | | | |
|---|---|---|---|---|---|---|---|---|
| | | | | | | | *Zm00001d021574* | MAPK activating protein |
| 73 | QTL-73 | 7 | Tieling | 166929352 | 9.33E-05 | 9.85% | *Zm00001d021950* | RNA-binding (RRM/RBD/RNP motifs) family protein |
| | | | | | | | *Zm00001d021951* | Spotted leaf protein 11 |
| | | | | | | | *Zm00001d021952* | Calcium-dependent lipid-binding (CaLB domain) family protein |
| 74 | QTL-74 | 7 | Tieling | 172484988 | 8.64E-05 | 11.11% | *Zm00001d022192* | Trehalose-6-phosphate phosphatase |
| | | | | | | | *Zm00001d022193* | Trehalose-6-phosphate phosphatase |
| 75 | QTL-75 | 9 | Tieling | 24651076 | 9.68E-05 | 10.37% | *Zm00001d045500* | 39S ribosomal protein L47 isoform 1 |
| | | | | | | | *Zm00001d045501* | Embryo sac development arrest 7 |
| | | | | | | | *Zm00001d045502* | NA |
| 76 | QTL-76 | 9 | Tieling | 129864633 | 2.97E-05 | 11.71% | *Zm00001d047433* | Histone deacetylase complex subunit SAP30 Sin3 binding domain-containing protein |
| | | | | | | | *Zm00001d047434* | Putative mediator of RNA polymerase II transcription subunit 26b |
| 77 | QTL-77 | 9 | Tieling | 153826566 | 8.74E-05 | 9.76% | *Zm00001d048280* | NA |
| | | | | | | | *Zm00001d048281* | Protein LURP-one-related 6 |
| | | | | | | | *Zm00001d048282* | Tubulin-tyrosine ligases |
| 78 | QTL-78 | 10 | Tieling | 135687334 | 7.69E-05 | 10.00% | *Zm00001d025996* | Ubiquitin carboxyl-terminal hydrolase family protein |
| | | | | | | | *Zm00001d025997* | Non-specific serine/threonine protein kinase |
| | | | | | | | *Zm00001d025998* | Ubiquitin carboxyl-terminal hydrolase |
| 79 | QTL-79 | 10 | Tieling | 140620275 | 9.90E-05 | 9.64% | *Zm00001d026191* | AP2-EREBP transcription factor |
| 80 | QTL-80 | 1 | Yuanyang | 263536337 | 2.16E-06 | 16.35% | *Zm00001d033467* | DWNN domain a CCHC-type zinc finger |
| | | | | | | | *Zm00001d033468* | Lactoylglutathione lyase / glyoxalase I family protein |
| | | | | | | | *Zm00001d033469* | Ferredoxin |
| 81 | QTL-81 | 1 | Yuanyang | 263584988 | 2.73E-06 | 14.78% | *Zm00001d033470* | Cytochrome P450 family 87 subfamily A polypeptide 2 |
| 82 | QTL-82 | 1 | Yuanyang | 263887558 | 3.84E-05 | 10.67% | *Zm00001d033478* | HSP20-like chaperones superfamily protein |
| | | | | | | | *Zm00001d033479* | Signal peptidase complex subunit 1 |
| | | | | | | | *Zm00001d033480* | 5-methyltetrahydropteroyltriglutamate--homocysteine methyltransferase |
| | | | | | | | *Zm00001d033481* | Protein FAR1-RELATED SEQUENCE 5 |
| 83 | QTL-83 | 1 | Yuanyang | 280483906 | 5.00E-05 | 11.10% | *Zm00001d033988* | Cytokinin-O-glucosyltransferase 1 |
| 84 | QTL-84 | 1 | Yuanyang | 280530679 | 5.00E-05 | 10.69% | *Zm00001d033989* | HXXXD-type acyl-transferase family protein |
| | | | | | | | *Zm00001d033990* | Bax inhibitor-1 family protein |
| | | | | | | | *Zm00001d033991* | Protein kinase superfamily protein |
| 85 | QTL-85 | 2 | Yuanyang | 42060673 | 3.96E-05 | 11.17% | *Zm00001d003373* | AAA+ ATPase domain-containing protein |
| | | | | | | | *Zm00001d003374* | NA |

| | | | | | | | | |
|---|---|---|---|---|---|---|---|---|
| | | | | | | | *Zm00001d003375* | OSJNBb0048E02.16-like protein |
| 86 | QTL-86 | 2 | Yuanyang | 154142006 | 8.17E-05 | 10.00% | *Zm00001d005019* | 4-coumarate--CoA ligase |
| 87 | QTL-87 | 2 | Yuanyang | 217963383 | 8.86E-05 | 10.22% | *Zm00001d006854* | ornithine carbamoyltransferase |
| | | | | | | | *Zm00001d006855* | Inter-alpha-trypsin inhibitor heavy chain-related |
| | | | | | | | *Zm00001d006856* | Pentatricopeptide repeat-containing protein mitochondrial |
| | | | | | | | *Zm00001d006857* | NA |
| 88 | QTL-88 | 3 | Yuanyang | 18650189 | 4.11E-05 | 10.58% | *Zm00001d039899* | PRPK |
| | | | | | | | *Zm00001d039900* | Probable zinc metalloprotease EGY2 chloroplastic |
| | | | | | | | *Zm00001d039901* | NA |
| | | | | | | | *Zm00001d039902* | ATP binding protein |
| 89 | QTL-89 | 3 | Yuanyang | 153388627 | 1.99E-05 | 11.61% | *Zm00001d042142* | Probable inactive purple acid phosphatase 16 |
| | | | | | | | *Zm00001d042143* | Glucan endo-13-beta-glucosidase homolog1 |
| | | | | | | | *Zm00001d042144* | High chlorophyll fluorescence 153 |
| 90 | QTL-90 | 3 | Yuanyang | 160487429 | 4.14E-05 | 12.33% | *Zm00001d042312* | Histidine kinase2 |
| | | | | | | | *Zm00001d042313* | Calmodulin-binding transcription activator 4 |
| 91 | QTL-91 | 3 | Yuanyang | 160557845 | 4.93E-05 | 10.60% | *Zm00001d042314* | CBS domain-containing protein CBSCBSPB1 |
| 92 | QTL-92 | 4 | Yuanyang | 193040531 | 4.95E-05 | 12.77% | *Zm00001d052557* | NA |
| | | | | | | | *Zm00001d052558* | Dirigent protein |
| 93 | QTL-93 | 5 | Yuanyang | 16264695 | 8.02E-05 | 11.00% | *Zm00001d013652* | Cationic amino acid transporter 4 vacuolar |
| | | | | | | | *Zm00001d013653* | Transcription regulator NOT2/NOT3/NOT5 family protein |
| 94 | QTL-94 | 5 | Yuanyang | 36483814 | 7.84E-05 | 10.14% | *Zm00001d014203* | F-box/kelch-repeat protein SKIP11 |
| | | | | | | | *Zm00001d014204* | Oxidoreductase |
| 95 | QTL-95 | 5 | Yuanyang | 48260848 | 4.27E-05 | 10.53% | *Zm00001d014458* | Glutamate receptor 3.4 |
| | | | | | | | *Zm00001d014459* | Cytochrome P450 superfamily protein |
| 96 | QTL-96 | 6 | Yuanyang | 168309048 | 9.89E-05 | 10.10% | *Zm00001d038987* | Auxin-responsive protein |
| | | | | | | | *Zm00001d038988* | hydroxyproline-rich glycoprotein family protein |
| | | | | | | | *Zm00001d038989* | Rop guanine nucleotide exchange factor 9 |
| | | | | | | | *Zm00001d038990* | Tumor protein-like protein |
| | | | | | | | *Zm00001d038991* | Protein MALE DISCOVERER 2 |
| 97 | QTL-97 | 7 | Yuanyang | 168609483 | 4.94E-05 | 10.60% | *Zm00001d022045* | tRNA/rRNA methyltransferase (SpoU) family protein |
| 98 | QTL-98 | 8 | Yuanyang | 96226720 | 6.86E-05 | 10.60% | *Zm00001d010031* | PIT1 |
| | | | | | | | *Zm00001d010032* | Pentatricopeptide repeat-containing protein mitochondrial |
| 99 | QTL-99 | 9 | Yuanyang | 102108865 | 6.37E-05 | 10.22% | *Zm00001d046678* | NA |

| | |
|---|---|
| *Zm00001d046679* | Potassium transporter |
| *Zm00001d046680* | Pentatricopeptide repeat-containing protein |
| *Zm00001d046681* | Probable glucomannan 4-beta-mannosyltransferase 9 |

Notes: All QTLs with overlapping QTL regions were categorized as a loci; Physical position of each SNP were based on B73 RefGen_V4; P value of the corresponding trait were calculated by Q+K model; The phenotypic variance were explained by the corresponding locus.
